# Clinical Advances in Cardiovascular Computed Tomography: From Present Applications to Promising Developments

**DOI:** 10.1007/s11886-024-02110-w

**Published:** 2024-08-20

**Authors:** Alexander Schulz, James Otton, Tarique Hussain, Tayaba Miah, Andreas Schuster

**Affiliations:** 1 Department of Cardiology and Pneumology, Georg-August University, University Medical Center, Göttingen, Germany; 2 South Western Sydney Clinical School, University of New South Wales, Sydney, NSW Australia; 3 Division of Imaging Sciences and Biomedical Engineering, King’s College London, London, UK; 4 Departments of Paediatrics, Southwestern Medical Center, University of Texas, Dallas, TX USA; 5 Department of Medicine, Cardiovascular Division, Beth Israel Deaconess Medical Center and Harvard Medical School, 330 Brookline Ave, Boston, MA 02215 USA; 6 FORUM Cardiology, Rosdorf, Germany

**Keywords:** Cardiovascular computed tomography, Cardiovascular imaging, Coronary artery disease, Structural heart disease, Diagnostic decision-making

## Abstract

**Purpose of the Review:**

This review aims to provide a profound overview on most recent studies on the clinical significance of Cardiovascular Computed Tomography (CCT) in diagnostic and therapeutic pathways. Herby, this review helps to pave the way for a more extended but yet purposefully use in modern day cardiovascular medicine.

**Recent Findings:**

In recent years, new clinical applications of CCT have emerged. Major applications include the assessment of coronary artery disease and structural heart disease, with corresponding recommendations by major guidelines of international societies. While CCT already allows for a rapid and non-invasive diagnosis, technical improvements enable further in-depth assessments using novel imaging parameters with high temporal and spatial resolution. Those developments facilitate diagnostic and therapeutic decision-making as well as improved prognostication.

**Summary:**

This review determined that recent advancements in both hardware and software components of CCT allow for highly advanced examinations with little radiation exposure. This particularly strengthens its role in preventive care and coronary artery disease. The addition of functional analyses within and beyond coronary artery disease offers solutions in wide-ranging patient populations. Many techniques still require improvement and validation, however, CCT possesses potential to become a “one-stop-shop” examination.

## Introduction

The application of groundbreaking non-invasive imaging techniques in the field of cardiovascular diseases is steadily increasing. Although transthoracic echocardiography remains the primary and most frequently used imaging modality in cardiovascular medicine, there are additional benefits of alternative non-invasive modalities, including cardiovascular computed tomography (CCT), cardiovascular magnetic resonance imaging (CMR), positron emission tomography (PET), and single-photon emission computed tomography (SPECT). Each imaging modality, characterized by unique advantages, is recommended for specific clinical situations based on these distinctive capabilities.

For CCT, hardware and software developments now allow for high-quality scans with low-dose radiation and very short scan times so that the major previous drawbacks associated with the negative effects of radiation are substantially mitigated [1].

Simultaneously with these technological advancements, there have been significant improvements in imaging quality, enabling the acquisition of intricate details during screening processes, valve repair procedures, evaluation of congenital heart diseases (CHD), and even in the differentiation of tissue composition in cardiomyopathies. Current guidelines now regard CCT as a critical preliminary step before invasive procedures, endorsing the use of CT imaging prior to echocardiography in patients presenting with chest pain [2]. In acute scenarios, CCT is now recommended as a class I recommendation to rule out coronary artery disease (CAD) in patients with out-of-hospital cardiac arrest (OHCA) and no signs of STEMI [3–5].

As techniques and applications are rapidly evolving, this review aims to provide an overview on the role of CCT in cardiovascular medicine including recent technical advances, and their potential clinical capabilities for state-of-the-art imaging with a particular focus on the trajectory of future developments (see Central Figure).

## Technical Considerations – From Hounsfield units to Multi-Energy Scanning

Prior to delving into the clinical applications and recent developments for specific diagnostic inquiries, it is imperative to consider some fundamental technical aspects concerning the hardware and software components of CCT.

Apart from patient characteristics and compliance (for instance, the ability to breath-hold during examinations), image quality in CCT is technically determined by parameters such as temporal, contrast, and spatial resolution [1].

### Temporal Resolution

For cardiac imaging in particular, temporal resolution carries significant importance due to the heart's perpetual motion, which generates motion artifacts. These artifacts are aggravated by respiratory motion. Moreover, temporal resolution assumes crucial significance when conducting functional evaluations of the heart or diagnosing CHD, especially in children who tend to have higher heart rates.

Temporal resolution is primarily determined by the gantry rotation time which reaches up to 250 ms in modern scanners [6, 7]. However, as the rotation speed cannot be increased without limitation, other technical developments are required to make further improvements. One innovation in CCT design was the introduction of Dual Source CT (DSCT), where a second X-ray tube and detector is installed within the CT scanner. By utilizing a second X-ray source, DSCT requires approximately one-quarter gantry rotation to enable image reconstruction [8, 9]. Modern 3rd generation DSCTs are able to acquire images at a temporal resolutions of 66 ms, which allows for imaging at much higher heart rates and even in the presence of arrhythmias at a reasonable spatial resolution [8]. Alternatively, DSCT may be used to provide a high-pitch single-heartbeat acquisition to scan the whole heart within a single cardiac cycle (i.e. flash-mode). Although subject to certain limitations with regard to heart rate, body habitus, and coronary calcification, this scan mode can provide imaging at low radiation doses (see Table 1). [19]

**Table 1:** Effective radiation dose of invasive and CT-coronary angiography

Procedure		Effective dose in mSv
Invasive coronary angiography		5—8 [10, 11 ]
Computed Tomography	Retrospective ECG-gating	9—16 [10]
	Prospective ECG-gating (“step-and-shoot”)	3—4 [10, 12 ]
	Prospective ECG-gating (high-pitch “Flash”-mode)	0.26—2 [12, 13 ]
	Coronary calcium scoring	0.26 – 0.76 [14, 15 ]
	Wide volume single heart beat acquisition	0.5—4 [16, 17 ]
	Ultra high resolution CT	5.4—7.4 [18]

Another important advancement was the introduction of multi-slice CT to increase the number of slices that can be acquired during a single rotation [20]. In turn, the volume of interest can be scanned faster and scans of the whole body can be performed within 1–2 s. [21] Wide volume detectors allow a volumetric imaging in one heartbeat without time delay.

### Spatial Resolution

The second major influencing factor of CCT is spatial resolution. As previously discussed, temporal resolution is vital for dose reduction, shorter scanning times, and functional analyses. Conversely, spatial resolution has a significant role in the primary application of CCT, which is morphological coronary imaging. This facet is essential in visualizing and quantifying intracoronary structures. Regular clinical CCT acquisition provides a resolution of approximately 0.25–0.5 mm while maintaining a satisfactory signal-to-noise ratio [18]. Initial utilisation of ultra-high resolution (UHR) CCT, with down to 150-200um resolution where made in a research environment [22]. UHR CT demonstrated higher positive and negative predictive values within calcified lesions and a more detailed quantification of in-stent dimension compared to regular CCT [23]. However, UHR CCT was associated with increased mean radiation doses (compare Table 1) [23, 24].

One method of increasing CT resolution is reducing the size of the detector elements (or increasing the slice number while maintaining detector width). However, increasing detector row numbers may be subject to a progressive deformation at the edge of the field of view, and increased scatter and loss of efficiency due to the relative increase in the area of the anti-scatter grid [21]. Additionally, higher gantry rotation speeds place multiple increased technical demands on CT detector design [1], [25].

Other methods to increase the resolution include rapid alternation of the X-ray tube electron beam focal point (‘flying focal spot’). This allows to obtain overlapping slices to increase the number of samples for a single projection and subsequently achieve sub-millimeter resolution [9, 26]. With the addition of advanced reconstruction methods it is possible to reconstruct thinner slices than acquired using standard retrospective helical acquisition techniques [27, 28]. As the increase in spatial resolution is generally traded for increased image noise, iterative reconstruction algorithms, and deep learning reconstruction methods have been developed to improve image quality and simultaneously reduce radiation [29–31].

### Contrast Resolution

While the identification of small lesions and detailed structures is a significant feature of CCT, the differentiation of plaque subtypes and specific plaque features is also crucial. These distinctions are significant because they are associated with cardiovascular events [32]. The identification of tissue subtypes within those structures is determined by contrast resolution, which is the last technical aspect with high importance to be discussed.

Spectral CT is able to identify and quantify different materials by calculating x-ray photon energy information by subtraction of images at different energy fields as originally proposed by Hounsfield [33].

A more recent development are photon counting detectors (PCD) which were clinically approved by the FDA in Sep. 2021 [34]. The major difference to conventional energy-integrating detectors (EID) is that with EID, the total energy from a large number of photons is registered within one detector element. In comparison, a PCD registers the interaction of individual photons and approximates the energy spectrum of an individual pixel [35]. To subtract different energy levels in EID, either a physically resolved (e.g. DSCT) or temporally resolved scan is performed to measure different energy levels [36]. For PCD, the detector is able to sort incoming photons in predefined energy “bins” by individually set thresholds within one acquisition [35]. This increases spatial resolution and material contrast within single pixels during a single scan and reduces artifacts [37]. Consequently, PCD reduce radiation dose as well as the required dose of contrast agents [38].

## Applications – Present and Future

**Fig. 1:**
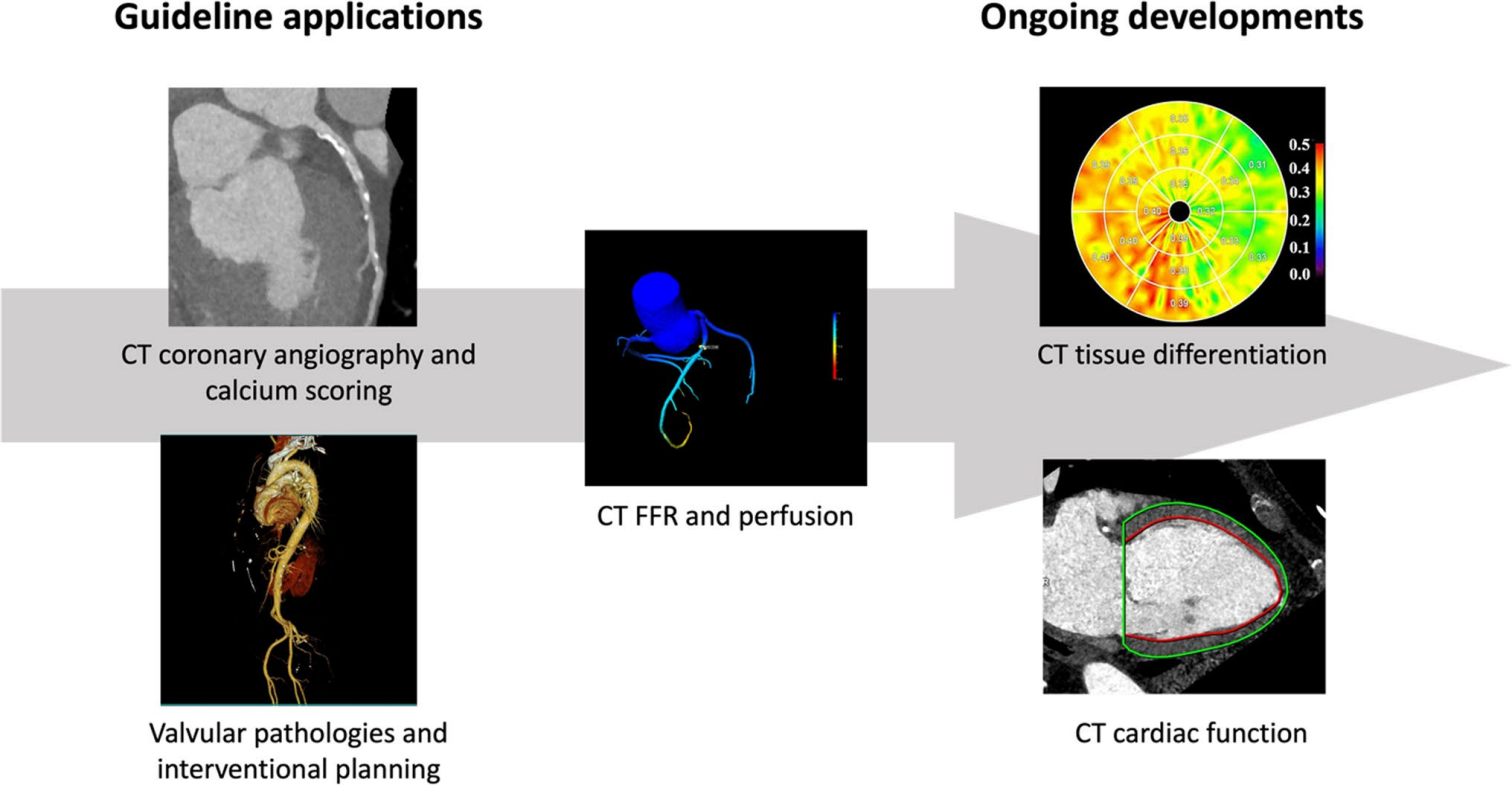
Central Figure: Cardiac CT applications in guidelines and current research. Cardiac CT is widely recognized and recommended in current guidelines. Recent developments potentially enable a broader application in various diseases offering a fast and effective alternative to established diagnostic pathways. (Parts of the image have been adapted from Yamada et al. [39] © CC-BY 4.0 https://creativecommons.org/licenses/by/4.0/.)

After providing insights into technical advances of CT imaging, the next chapter is dedicated to a brief introduction of CCT applications in cardiovascular medicine including ongoing developments (Fig. 1).

### Coronary Stenosis, Anomaly, and Ischemia – from CT-Angiography to CT-FFR and Perfusion

#### CCTA and Calcium Scoring

**Fig. 2:**
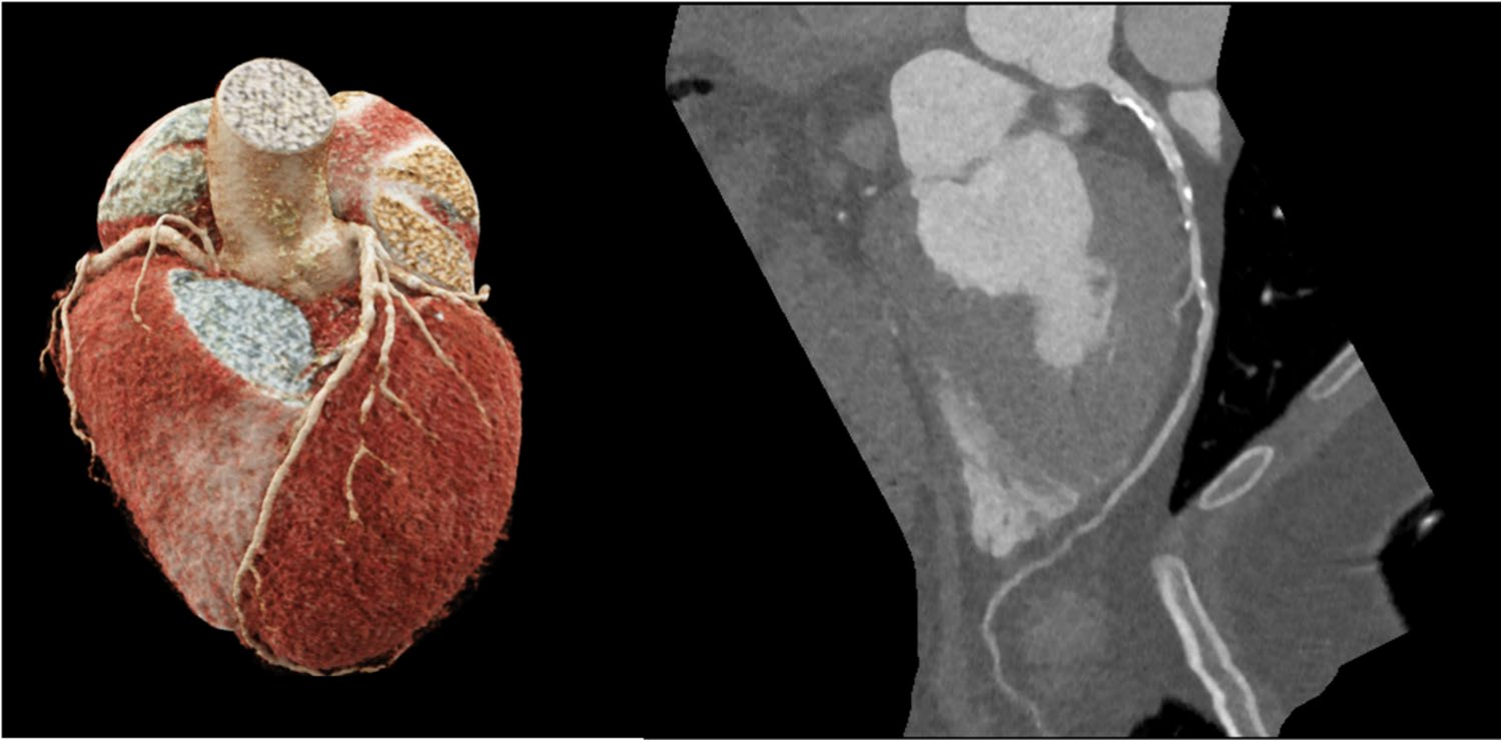
CT coronary angiography. The image on the left shows a 3D model of the coronary arteries. The right image displays a radial series of the left anterior descending artery of the same patient with calcified and non-calcified plaque in the proximal segment

Isotropic resolution allows for reconstruction in any orientation to depict coronary artery anatomy, anomalies and stenosis with high detail in 3D visualizations (see Fig. 2). Furthermore, CCT can provide sophisticated plaque characterization based on levels of calcification and adverse geometric features of plaques [40]. The latest scanner generations, especially using PCD technology, even allow for an accurate description of coronary artery in-stent re-stenosis [41].

Next to coronary computed tomography angiography (cCTA) coronary artery calcium scoring has emerged as an easy-to-use non-invasive parameter for the atherosclerotic burden and adds incremental prognostic value and safety to regular cCTA [42]. Novel applications include virtual non-contrast spectral CT imaging techniques, that allow calcium scoring with very low radiation doses (see Table 1) [43] and the estimation of the calcium score from contrast cCTA without the need for an additional scan [44].

#### CT-FFR

The reference standard for quantifying the hemodynamic relevance of coronary narrowings are invasive measurements of either fractional flow reserve (FFR) [45], to calculate the hyperemic reserve of a vessel during pharmacologically induced vasodilation or alternatively during the wave-free period using the instantaneous wave-free ratio (IFR) [46, 47]. However, using CCT imaging, an equivalent to the invasive approach can be offered with high per-vessel and per-patient agreement in published head-to-head comparisons [48, 49].

**Fig. 3:**
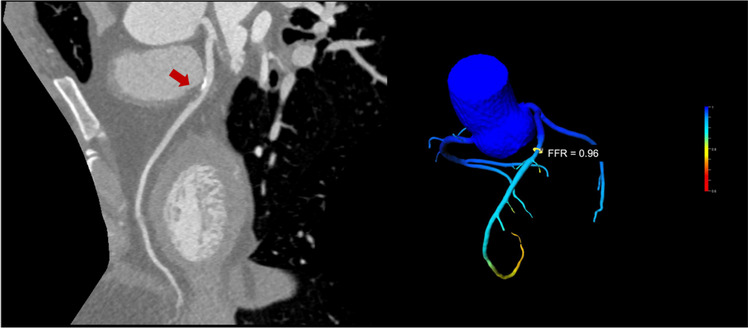
Functional testing using CT-FFR. The image on the left shows an intermediate stenosis of the left anterior descending artery by a mixed plaque. Using CT-FFR as shown on the right, it was possible to exclude haemodynamic relevance of the stenosis

CT-FFR measurements allow for calculation of blood flow over the stenosis using computational fluid dynamics or machine learning approaches (see Fig. 3) [50]. However, this technical approach is highly dependent on the image quality and segmentation of the coronary arteries.

#### CT-Perfusion

Guidelines recommend functional imaging options like stress-echocardiography, PET/SPECT, or CMR to detect wall-motion abnormalities or reduced perfusion in viable tissue [47]. CCT multidetector perfusion imaging during adenosine stress has emerged as a potential alternative for functional perfusion assessments [51–53]. However, in comparison to invasive FFR quantification, CT-Perfusion imaging revealed only a moderate accuracy to identify perfusion defects associated with ischemia. Notwithstanding, in combination with cCTA, CCT-Perfusion may allow to identify or exclude relevant ischemia within the myocardium subtended by a stenosed artery [54, 55, 56]﻿.

### Cardiomyopathies – Volumes and Tissue from CMR to CT

#### Cardiac Volumes

**Fig. 4:**
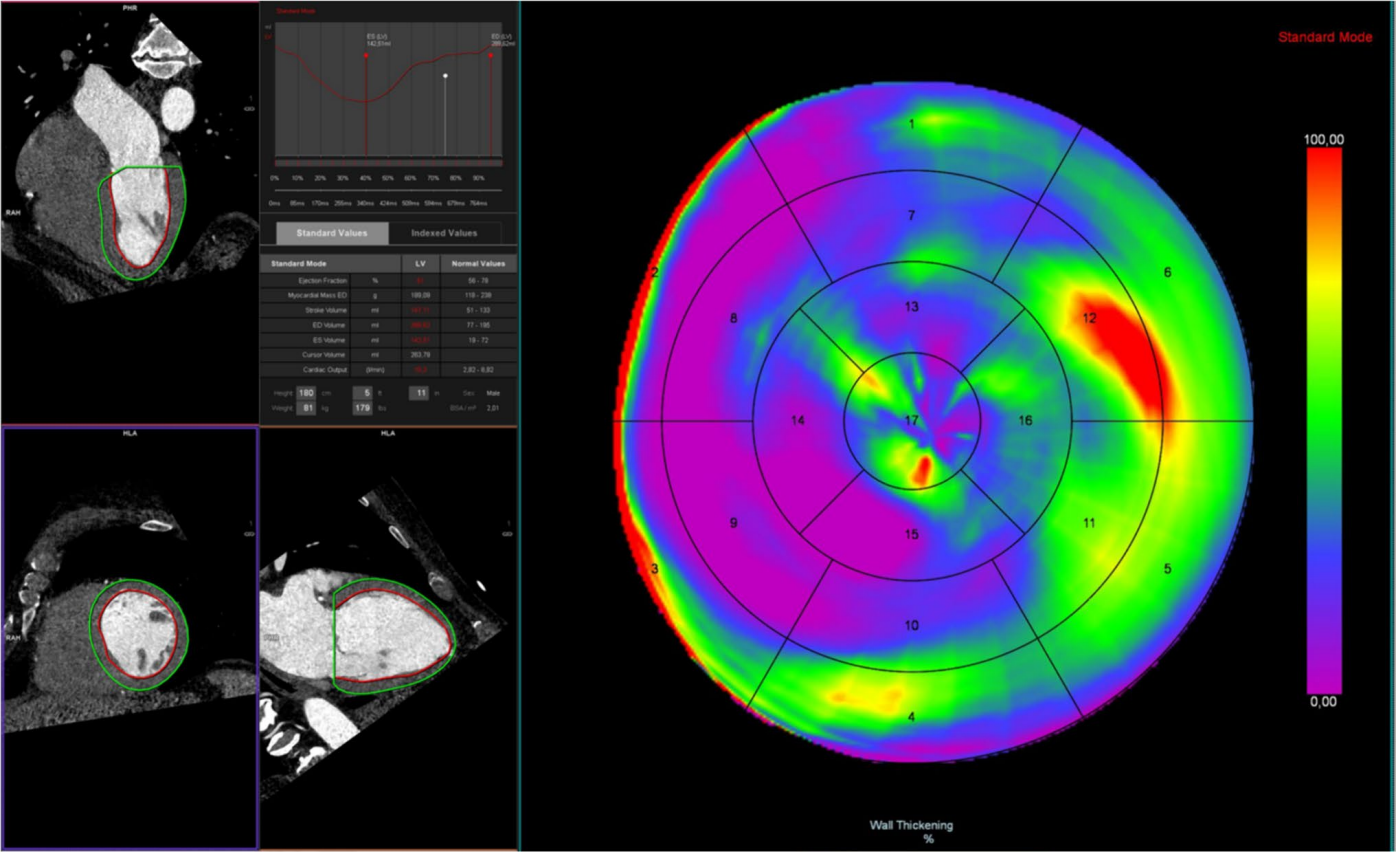
CT-derived volumetry and functional analysis. The images on the left describe the process of CT-based volumetry using endo- and epicardial contouring. Functional assessments allow the quantification of global cardiac function as well as in-depth visualization of wall-motion abnormalities as shown by the bullet plot on the right

Initial studies of volume quantification using CCT revealed overall good feasibility with varying but reasonable agreement with CMR as the reference standard. Mostly, a trend of an overestimation of volumes and underestimation of the ejection fraction (EF) was observed [57, 58]. However, with the development of multi-slice CT later studies could prove an increased accuracy of measurements of left ventricular EF compared to CMR and transthoracic echocardiography [59] and a concomitant assessment of right ventricular function [60]. An example of CT-based volumetric and functional measurements of the left ventricle is shown in Fig. 4.

More recently, even CT-derived strain measurements have been shown to be possible [61] and provided comparable measurements to CMR [62].

#### Tissue Characterization

In CCT, current developments address the assessment of myocardial edema and the extra cellular volume as an index of diffuse myocardial fibrosis.

Focal myocardial edema can be sufficiently assessed, as low Hounsfield units indicate water as the main component of the edema. Comparisons to T2-weighted CMR imaging using unenhanced DSCT imaging demonstrated good agreement between both methods [63].

**Fig. 5:**
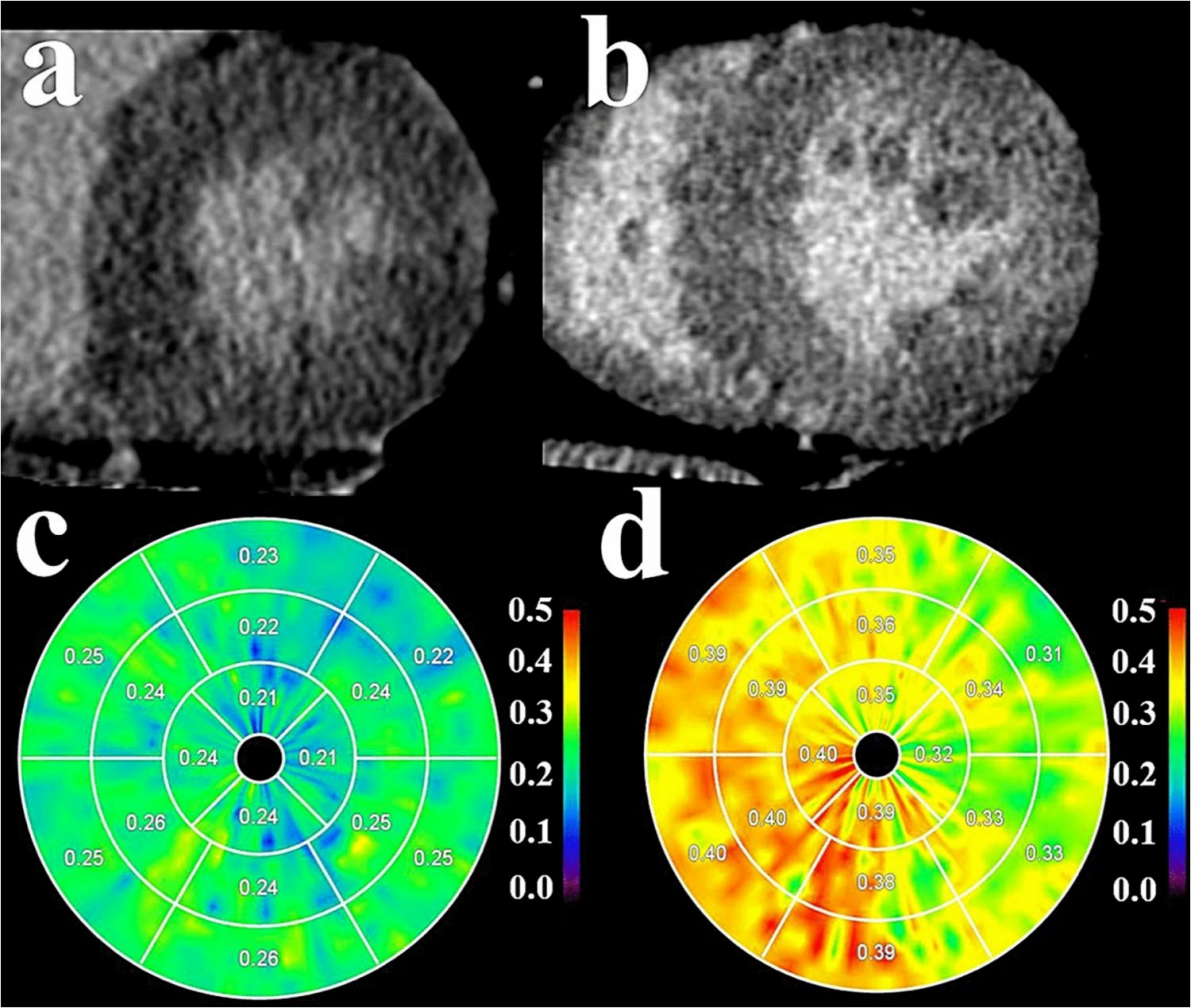
Calculation of ECV using CT. Delayed enhancement images and polar ECV maps of a patient with normal ECV (**a** and **c**) and a patient with increased ECV (**b** and **d**). ECV can be calculated by a subtraction image of a pre-contrast and a delayed enhancement post-contrast image. (By Yamada et al. [39] © CC-BY 4.0 https://creativecommons.org/licenses/by/4.0/.)

For quantification of the extra cellular volume (ECV) in CCT, measurements are performed by the creation of a subtraction image from pre- and post-contrast acquisitions. Using newer generations of dual-energy PCTs, ECV can be derived from a single delayed acquisition with similar accuracy and lower radiation doses (see Fig. 5) [64]. Even though the determination of diffuse myocardial abnormalities is very challenging considering the low contrast resolution of CCT, validation of current methods against CMR showed overall good agreement [65].

### Structural Heart Disease – from Valvular Pathologies to 4D Flow

CCT allows for spatially highly resolved imaging of the valves including morphological details such as the leaflets, and for the mitral valve, chordae, or papillary muscles. In contrast to echocardiography, valve assessment is not based on hemodynamical pressure and velocity gradients, but on the direct planimetry of the aortic orifice during mid systole.

The highly detailed visualization of the valves is an increasing desire for the calculation of hemodynamics and cardiovascular flow. CCT requires the use of a contrast bolus for calculations of cardiovascular bloodflow [66] or to obtain transvalvular pressure gradients to complement the detailed morphological imaging with functional and physiological data to enhance valvular assessments [67].

First implementations of algorithms using computational fluid dynamics were even able to analyze 4D intracardiac flow patterns [68, 69]. Even though those techniques are still in their infancy**,** calculation of 4D data in cardiovascular assessments has received considerable attention in general and is also likely to grow in CCT.

## Clinical Perspective

Novel imaging capabilities of CCT have enforced a widespread use across various cardiac diseases. Considering the high performance of CCT regarding diagnosis, prognostication, and screening particularly of CAD and increasing guideline recommendations, we have detailed current and future clinical value in the following section.

### Clinical Value in Prevention

Non-contrast coronary artery calcium scoring at the start of the scan offers accurate coronary screening and crucial predictive information [70, 71] with high therapeutic impact [72].

CCT is able to detect coronary artery plaque at an early stage [70]. Even though plaques might be non-obstructive, their prognostic importance has been well demonstrated [73]. In particular, in mild nonobstructive plaques with 0–49% stenosis, coronary calcification predicted mortality significantly better than conventional risk assessment by the Framingham risk score [74].

The Multi-Ethnic Study of Atherosclerosis has established percentiles for four major racial and ethnic groups, discovering that a doubling of coronary artery calcification elevates the risk of major coronary events from 15 to 35% percent [75]. Moreover, the total plaque burden was found to be a major risk predictor of cardiovascular events and death independently of the presence of stenosis. This led to an amendment in the revised CAD-RADS 2.0 criteria, now including the total plaque burden as a new category for risk assessment [76, 77].

From a therapeutical perspective, an early identification of coronary plaques can lead to an early start of statin therapy to stabilize plaques and reduce atherosclerotic risk [78]. Next to statins non-invasive coronary artery calcium scoring also governs the use of aspirin for primary prevention as patients with more severe coronary artery calcifications are more likely to benefit from statins and aspirin [79]. Patients with a risk for CAD greater than  10% as indicated by the Framingham risk score and a calcium score between 1–99 were shown to benefit from medication already, since the number needed to treat falls below the number needed to harm. In patients with a calcium score greater than 100,  this additional benefit of medical prevention was observed for all patients, irrespective of their Framingham risk score [80]. This impact of CT-based risk screening on primary preventive measures  has not been shown for any other modality, underlining the importance of CCT in preventive cardiovascular care.

Counterintuitively to the findings on medical therapy, a recent study found that lifelong endurance training was associated with higher plaque burden as detected by CCT despite the presumed healthier lifestyle of athletes [81]. Notwithstanding, the study found that the plaques athletes, may arise from wall stress during enduring exercise and are as stable as  plaques during statin therapy. A longitudinal follow-up of this study cohort would be required to further enhance our understanding of the importance of different forms of exercise in cardiovascular prevention after plaque detection by CCT.

### Clinical Value in Coronary Artery Disease

CTA has experienced a major boost after consideration and valuation as a class I recommendation within the 2016 NICE guidelines, 2019 ESC guidelines, 2021 AHA/ACC guidelines for chest pain and chronic coronary syndrome [2, 47, 82], and the 2022 guidelines for ventricular arrhythmias and sudden cardiac death [5].

Due to high sensitivity, it is being recommended for patients with low- to intermediate-risk for chronic CAD to rule-out CAD due to its high negative predictive value [47, 83–85]. In turn, the lower specificity may require functional testing in patients with higher-risk constellations [47]. Nevertheless, when assessing its utility in routine clinical practice, CCT was validated as an important alternative to functional testing in the context of the PROMISE trial [86] and as an effective tool for additional clinical decision-making in the SCOT-HEART trial [87]. Non-inferiority of CCT as an initial diagnostic step compared to invasive coronary angiography has been shown by multiple studies [87–89].

Even in the very early stages of atherosclerosis, CCT yields capabilities for in-depth risk prediction using the fat attenuation index by quantifying perivascular adipose tissue. This may point to anti-inflammatory therapy interventions and allow for disease activity monitoring [90]. As such, it offers a cost-effective alternative in patients with stable chest pain symptoms [91] while helping to avoid unnecessary coronary angiographies [87, 92].

For clinical reporting the use of the Coronary Artery Disease – Reporting and Data System (CAD-RADS) is recommended by international societies [93]. In addition to a standardized framework for a comprehensive reporting on plaque burden, stenosis and specific modifiers, CAD-RADS provides dedicated recommendations on further diagnostic and therapeutic steps.

Other studies investigated CCT in acute coronary syndromes and found that safe discharge of patients with low- to intermediate-risk for acute coronary syndrome can be provided following CCT assessment. Strategies involving CCT in a triage system at the emergency department for patients with suspected acute coronary syndrome resulted in a more rapid and safe discharge with similar or lower total costs of treatment [94–96]. However, potential downstream testing and total radiation exposure increased, indicating the need for precise clinical assessments when using CCT for decision-making at the emergency department [96].

To address the lower specificity of CCT, further functional testing of hemodynamic relevance of stenoses have been introduced by CT perfusion or CT FFR within the same modality [97]. Testing for ischemia using CCT has now been included in the revised CAD-RADS II criteria as an additional modifier “I” and is suggested in patients with moderate to high (50–90%) stenosis i.e. CAD-RADS 3 and 4A when technically available [93].

Compared to non-invasive CMR-Perfusion, isolated CCT-Perfusion is inferior, even though its combination with anatomical cCTA increases accuracy and results in similar global performance [55]. While CT-FFR offers an alternative functional assessment without the need of additional radiation, it is important to note that high standardization is recommended according to SCCT and RSNA guidelines. [97, 98] This is mainly due to the rate of undiagnostic results that are still described to occur between 7–12% of scans, which may explain why this technique has not yet fully been adopted clinically [99, 100]. In addition, CT-FFR suffers from a “grey area” between 0.76–0.8 potentially requiring referral for invasive diagnostics [93]. Therefore, guidelines consider CT-FFR currently only as an amendment to regular cCTA [97, 98]. Bearing these limitations in mind, CT-FFR has been demonstrated to increase the specificity and positive predictive value of CCT compared to regular cCTA [101]. Furthermore, it has been suggested that CT-FFR could potentially reduce costs compared to invasive procedures, taking into account that pressure wires are single-use items and must be disposed of post-measurements. Interestingly, no total cost reduction could be found compared to standard clinical care, but CT-FFR was able to significantly reduce the number of invasive coronary angiography [102].

### Clinical Value in Cardiomyopathies

For assessments of the impact of plaque burden and degree of coronary stenosis on the myocardium, the quantification of myocardial function and tissue viability is of equal significance. In CAD with a subsequent risk of heart failure and development of scar tissue, these assessments can guide medical and device therapy. Even though transthoracic echocardiography is the most widely used diagnostic tool for quantification of function and CMR remains the mainstay for tissue characterization, it would be of interest to obtain additional data on cardiac function and remodeling within the same scan and modality.

Even though volumetric assessments can be easily obtained from CCT scans comprising the whole cardiac cycle and volume, they still require a dedicated protocol, as a post hoc analysis from a scan solely focusing on coronary arteries is not possible yet. Furthermore, volumetric scans come with the trade-off of increased doses of radiation and contrast agents [103] which currently makes them less applicable in clinical routine. Several approaches have been made to overcome the need for contrast agent application and to retrospectively analyze data from non-contrast images, however, those techniques require further validation for broader applicability in the future [104–106].

The domain of tissue characterization in cardiac CT has been established more recently and offers promising prospects for future applicability. Even though the range of normal values of ECV is still a major impediment to clinical application [107], ECV plays an important role in patients with aortic stenosis [108]. Cardiac amyloidosis is characterized by a significantly increased ECV and co-present in 15–30% of aortic stenosis cases, which can theoretically be diagnosed from regular planning CTs for TAVR. In this matter, studies already found that the calculation of CT-derived ECV during routine TAVR evaluation is able to depict cardiac amyloidosis with high accuracy and provides information on the degree of infiltration [109].

While CMR will most likely remain superior to CCT for tissue characterization, CCT may be considered as an alternative in the presence of contraindications to CMR [110, 111]. Especially in patients with pacemakers, artifact compensation in CT is more robust compared to CMR, even though CMR imaging quality in these patient groups has improved in the past years.

### Clinical Value in Valvular and Congenital Heart Disease

**Fig. 6:**
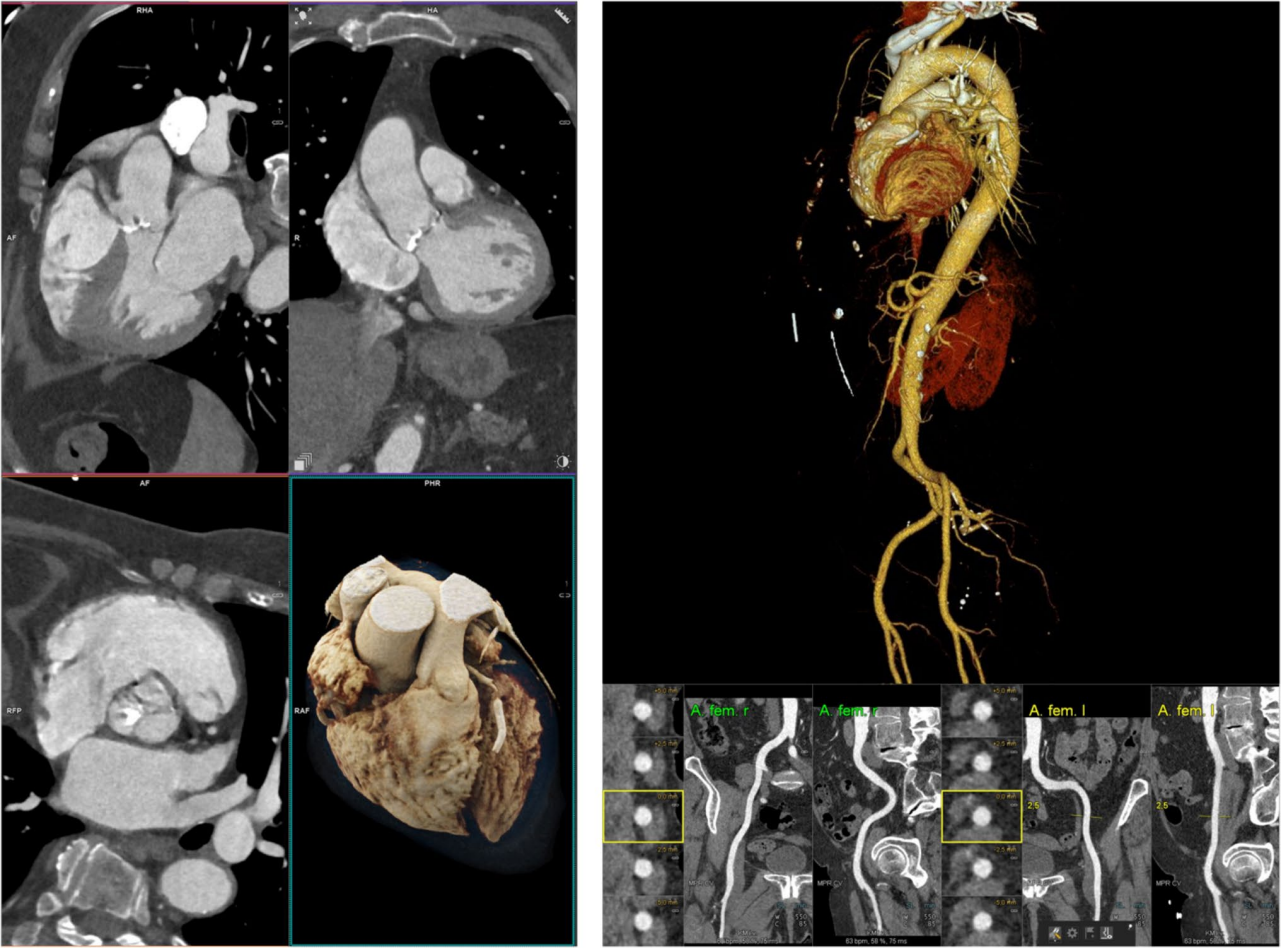
Transcatheter aortic valve replacement (TAVR) planning using CT. The images on the left delineate the measurement of the aortic annulus prior to TAVR in 3 different orientations of the aortic valve. The right image shows the access path via the right (A. fem. r) and left femoral artery (A. fem. l)

In clinical routine, CCT is mostly applied for sizing and selection of the optimal prosthesis as part of the preparation of invasive procedures, including the visualization of the access route for the aortic valve along the femoral arteries and descending aorta within the same examination (see Fig. 6) [112, 113, 114]﻿.

In addition, CCT was recommended by current ESC guidelines as flow-independent method of choice for advanced decision-making in patients with borderline findings in aortic stenosis using the assessment of aortic valve calcification [112, 115]. The distribution of calcifications along the aortic annulus can be visualized using CCT which helps to identify adverse features indicating a more complicated intervention, periprocedural risks [116, 117] or even an increased risk for postprocedural LVOT obstruction [118, 119]. Those prospects for an enhanced risk prediction extended the value of CCT beyond conventional procedural planning [120].

Furthermore, CCT can be used to determine reversible causes of valvular disease such as thrombosis or endocarditis if transthoracic and transesophageal echocardiography is inconclusive [112] and was now endorsed by the 2023 ESC guidelines more extensively [121].

The utility of dynamic CCT in the setting of infective endocarditis has been shown to be particularly important if cardiac devices are present such as in the setting of right ventricle to pulmonary artery conduits, which are in an anterior location that is not easily imaged on trans-esophageal echocardiography [122].

**Fig. 7:**
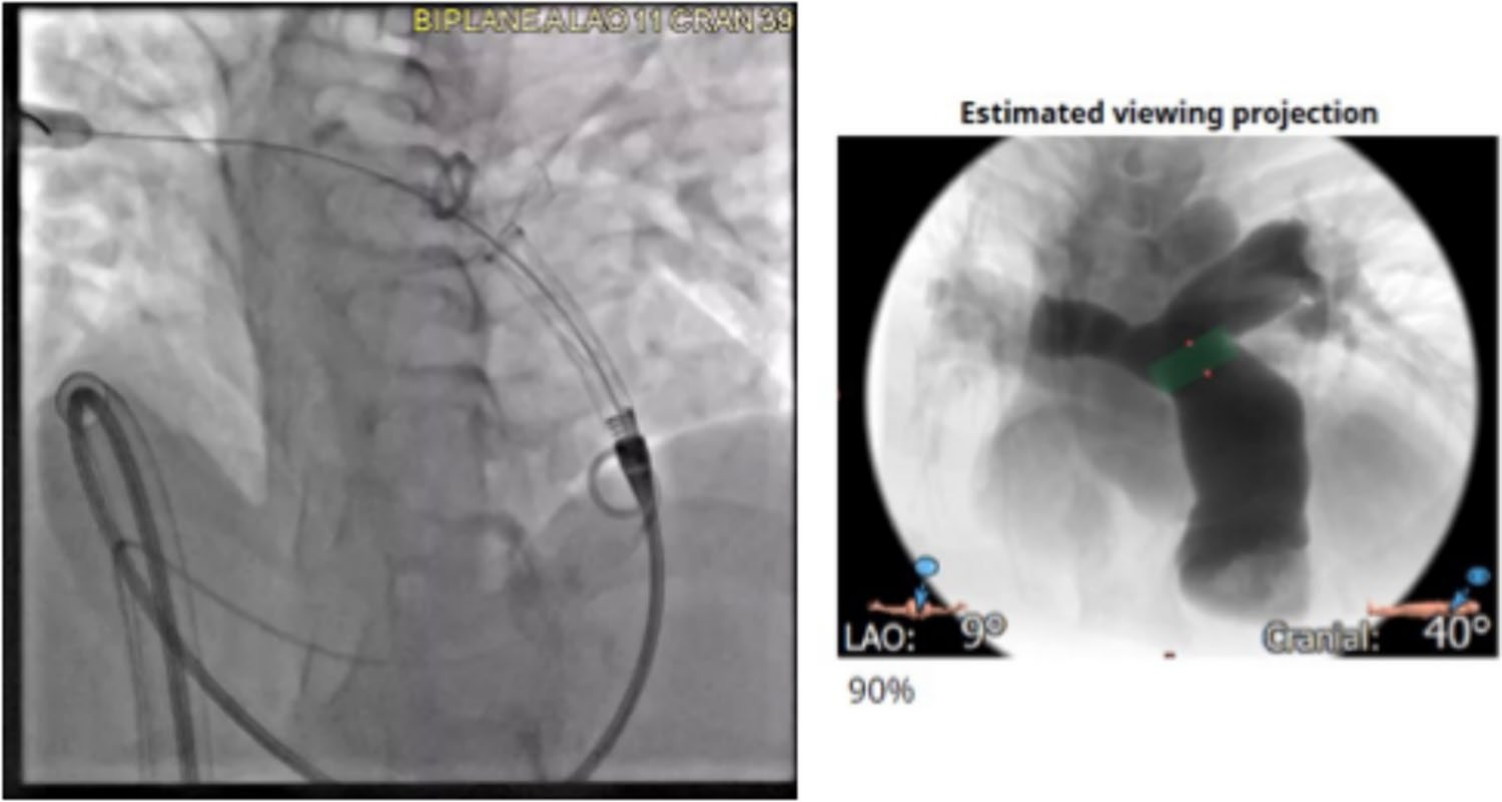
Shows the estimated optimal angiographic projection using the CT images on the right. The estimated distal landing zone shown in **green**. On the left panel, the actual angiographic deployment is shown taking benefit from CT-based predictions

Similarly to valvular diseases in adults, CCT has become the modality of choice for planning valvular device suitability in CHD for the new generation of percutaneously implanted large RVOT pulmonary valve systems [123]. Fig. 7 shows the ability of CCT not only to predict device choice and to define its landing zone but also the ability to provide the estimated angiographic projection.

But also considering other CHD such as coronary anomalies, it has also been shown that cCCT is able to reproducibly measure the proximal dimension of anomalous aortic origin of a coronary artery and so, may be helpful in risk prediction [124]. Another important advantage of CCT in CHD is the consistent threshold characteristics which are particularly advantageous for volume rendering and 3D printing of CHD. This approach has been shown to be able to depict complex anatomy for improved interventional and surgical planning [125].

## Conclusion

CCT has become an irreplaceable instrument in contemporary medicine. Its capabilities in anatomical imaging supply crucial information in an array of heart diseases, while additional functional assessments significantly broaden the range of CCT applications. Some of these techniques still need to establish their additional value in clinical contexts and require further validation in large, multicentric trials. If the hardware and software refinements keep up to speed, CCT possesses significant potential to become a "one-stop-shop" non-invasive imaging method, not only for CAD but also for other diagnostic applications.

## Data Availability

No datasets were generated or analysed during the current study.

## Abbreviations

CCT: Cardiovascular computed tomography
CMR: Cardiovascular magnetic resonance imaging
PET: Positron emission tomography
SPECT: Single photon emission computed tomography
DSCT: Dual source CT
UHR: Ultra high resolution
PCD: Photon counting detector
EID: Energy-integrating detector
FFR: Fractional flow reserve
Ccta: Coronary computed tomography angiography
IFR: Instantaneous wave free ratio
TAVR: Transcatheter aortic valve replacement
CHD: Congenital heart disease
CAD: Coronary artery disease
LGE: Late gadolinium enhancement
ECV: Extracellular volume
LVOT: Left ventricular outflow tract
EF: Ejection fraction

## References

[1] Toia P, et al. Technical development in cardiac CT: current standards and future improvements—a narrative review. Cardiovasc Diagn Ther. 2020;10:2018–35. 33381441 10.21037/cdt-20-527 PMC7758763

[2] Gulati M, et al. 2021 AHA/ACC/ASE/CHEST/SAEM/SCCT/SCMR Guideline for the Evaluation and Diagnosis of Chest Pain: A Report of the American College of Cardiology/American Heart Association Joint Committee on Clinical Practice Guidelines. J Am Coll Cardiol. 2021;78:e187–285. 34756653 10.1016/j.jacc.2021.07.053

[3] Lemkes JS, et al. Coronary Angiography after Cardiac Arrest without ST-Segment Elevation. N Engl J Med. 2019;380:1397–407. 30883057 10.1056/NEJMoa1816897

[4] Desch S, et al. Angiography after Out-of-Hospital Cardiac Arrest without ST-Segment Elevation. N Engl J Med. 2021;385:2544–53. 34459570 10.1056/NEJMoa2101909

[5] Zeppenfeld K, et al. 2022 ESC Guidelines for the management of patients with ventricular arrhythmias and the prevention of sudden cardiac death: Developed by the task force for the management of patients with ventricular arrhythmias and the prevention of sudden cardiac death of the European Society of Cardiology (ESC) Endorsed by the Association for European Paediatric and Congenital Cardiology (AEPC). Eur Heart J. 2022;43:3997–4126. 36017572 10.1093/eurheartj/ehac262

[6] Schmidt B, Grant K, Flohr TG, Allmendinger T. Cardiac CT Platforms: State of the Art. in *CT of the Heart* (ed. Schoepf, U. J.) 51–67 (Humana Press, 2019). 10.1007/978-1-60327-237-7_6

[7] Ohnesorge, B. Future Technical Developments in Cardiac CT. in *Multi-slice and Dual-source CT in Cardiac Imaging: Principles — Protocols — Indications — Outlook* (eds. Ohnesorge, B. M., Flohr, T. G., Becker, C. R., Knez, A. & Reiser, M. F.) 327–358 (Springer, 2007). 10.1007/978-3-540-49546-8_22

[8] Petersilka M, Bruder H, Krauss B, Stierstorfer K, Flohr TG. Technical principles of dual source CT. Eur J Radiol. 2008;68:362–8. 18842371 10.1016/j.ejrad.2008.08.013

[9] Lin E, Alessio A. What are the basic concepts of temporal, contrast, and spatial resolution in cardiac CT? J Cardiovasc Comput Tomogr. 2009;3:403–8. 19717355 10.1016/j.jcct.2009.07.003 PMC4752333

[10] Gerber TC, Kantor B, McCollough CH. Radiation Dose and Safety in Cardiac Computed Tomography. Cardiol Clin. 2009;27:665–77. 19766923 10.1016/j.ccl.2009.06.006 PMC2749002

[11] de AraújoGonçalves P, et al. Effective radiation dose of three diagnostic tests in cardiology: Single photon emission computed tomography, invasive coronary angiography and cardiac computed tomography angiography. Rev Port Cardiol Engl Ed. 2013;32:981–6. 10.1016/j.repc.2013.05.005 24287017

[12] Deseive S, et al. Image quality and radiation dose of a prospectively electrocardiography-triggered high-pitch data acquisition strategy for coronary CT angiography: The multicenter, randomized PROTECTION IV study. J Cardiovasc Comput Tomogr. 2015;9:278–85. 25926015 10.1016/j.jcct.2015.03.001

[13] Zheng M, et al. Low-Concentration Contrast Medium for 128-Slice Dual-Source CT Coronary Angiography at a Very Low Radiation Dose Using Prospectively ECG-Triggered High-Pitch Spiral Acquisition. Acad Radiol. 2015;22:195–202. 25457735 10.1016/j.acra.2014.07.025

[14] Bechtiger FA, et al. Risk stratification using coronary artery calcium scoring based on low tube voltage computed tomography. Int J Cardiovasc Imaging. 2022;38:2227–34. 37726457 10.1007/s10554-022-02615-x PMC10509109

[15] Hecht HS, et al. Low- vs. standard-dose coronary artery calcium scanning. Eur Heart J Cardiovasc Imaging. 2016;16:358–63. 10.1093/ehjci/jeu218 25381303

[16] Utsunomiya D, et al. Evaluation of appropriateness of second-generation 320-row computed tomography for coronary artery disease. Springerplus. 2015;4:109. 25793150 10.1186/s40064-015-0866-1 PMC4359191

[17] Chen MY, Shanbhag SM, Arai AE. Submillisievert Median Radiation Dose for Coronary Angiography with a Second-Generation 320–Detector Row CT Scanner in 107 Consecutive Patients. Radiology. 2013. 10.1148/radiol.13122621. 23340461 10.1148/radiol.13122621 PMC3606544

[18] Kwan AC, Pourmorteza A, Stutman D, Bluemke DA, Lima JAC. Next-Generation Hardware Advances in CT: Cardiac Applications. Radiology. 2021;298:3–17. 33201793 10.1148/radiol.2020192791 PMC7771994

[19] Meinel FG, Renker M (2019) Coronary CT angiography for screening, risk stratification, and management of asymptomatic patients: state of the evidence in Schoepf, U. J. Contemporary medical imaging. *CT of the Heart: Second Edition*. Humana Totowa, 2019 pp 739-745. https://doi.org/10.1007/978-1-60327-237-7_58

[20] Flohr T, Ohnesorge B (2007) Multi-slice CT technology. In: Ohnesorge B, Flohr T, Becker CR, Knez A, Reiser MF (eds) Multi-slice and dual-source CT in cardiac imaging. Principles – Protocols – Indications – Outlook, 2nd edn. Springer, Berlin, pp 41–69

[21] Flohr TG, Raupach R, Bruder H. Cardiac CT: how much can temporal resolution, spatial resolution, and volume coverage be improved? J Cardiovasc Comput Tomogr. 2009;3:143–52. 19527890 10.1016/j.jcct.2009.04.004

[22] Hata A, et al. Effect of Matrix Size on the Image Quality of Ultra-high-resolution CT of the Lung: Comparison of 512 × 512, 1024 × 1024, and 2048 × 2048. Acad Radiol. 2018;25:869–76. 29373211 10.1016/j.acra.2017.11.017

[23] Motoyama S, et al. Ultra-High-Resolution Computed Tomography Angiography for Assessment of Coronary Artery Stenosis. Circ J Off J Jpn Circ Soc. 2018;82:1844–51. 10.1253/circj.CJ-17-1281 29743388

[24] Takagi H, et al. Diagnostic performance of coronary CT angiography with ultra-high-resolution CT: Comparison with invasive coronary angiography. Eur J Radiol. 2018;101:30–7. 29571798 10.1016/j.ejrad.2018.01.030

[25] Goldman LW. Principles of CT and CT technology. J Nucl Med Technol. 2007;35:115–28. 17823453 10.2967/jnmt.107.042978

[26] Halliburton S, et al. State-of-the-art in CT hardware and scan modes for cardiovascular CT. J Cardiovasc Comput Tomogr. 2012;6:154–63. 22551595 10.1016/j.jcct.2012.04.005 PMC3677072

[27] Kalisz K, et al. Artifacts at Cardiac CT: Physics and Solutions. Radiogr Rev Publ Radiol Soc N Am Inc. 2016;36:2064–83. 10.1148/rg.2016160079 27768543

[28] Kumamaru KK, Hoppel BE, Mather RT, Rybicki FJ. CT Angiography: Current Technology and Clinical Use. Radiol Clin North Am. 2010;48:213–35. 20609871 10.1016/j.rcl.2010.02.006 PMC2901244

[29] Willemink MJ, et al. Iterative reconstruction techniques for computed tomography Part 1: technical principles. Eur Radiol. 2013;23:1623–31. 23314600 10.1007/s00330-012-2765-y

[30] Halliburton SS, Tanabe Y, Partovi S, Rajiah P. The role of advanced reconstruction algorithms in cardiac CT. Cardiovasc Diagn Ther. 2017;7:527–38. 29255694 10.21037/cdt.2017.08.12 PMC5716948

[31] Wang R, et al. Image quality and radiation dose of low dose coronary CT angiography in obese patients: sinogram affirmed iterative reconstruction versus filtered back projection. Eur J Radiol. 2012;81:3141–5. 22578834 10.1016/j.ejrad.2012.04.012

[32] Virmani R, Kolodgie FD, Burke AP, Farb A, Schwartz SM. Lessons from sudden coronary death: a comprehensive morphological classification scheme for atherosclerotic lesions. Arterioscler Thromb Vasc Biol. 2000;20:1262–75. 10807742 10.1161/01.atv.20.5.1262

[33] Hounsfield GN. Computerized transverse axial scanning (tomography) Description of system. Br J Radiol. 1973;46:1016–22. 4757352 10.1259/0007-1285-46-552-1016

[34] Commissioner, O. of the. FDA Clears First Major Imaging Device Advancement for Computed Tomography in Nearly a Decade. *FDA* https://www.fda.gov/news-events/press-announcements/fda-clears-first-major-imaging-device-advancement-computed-tomography-nearly-decade (2021).

[35] Willemink MJ, Persson M, Pourmorteza A, Pelc NJ, Fleischmann D. Photon-counting CT: Technical Principles and Clinical Prospects. Radiology. 2018;289:293–312. 30179101 10.1148/radiol.2018172656

[36] Taguchi K, Iwanczyk JS. Vision 20/20: Single photon counting x-ray detectors in medical imaging. Med Phys. 2013;40:100901. 24089889 10.1118/1.4820371 PMC3786515

[37] Alvarez RE, Macovski A. Energy-selective reconstructions in X-ray computerized tomography. Phys Med Biol. 1976;21:733–44. 967922 10.1088/0031-9155/21/5/002

[38] Roessl E, Proksa R. K-edge imaging in x-ray computed tomography using multi-bin photon counting detectors. Phys Med Biol. 2007;52:4679–96. 17634657 10.1088/0031-9155/52/15/020

[39] Yamada A, et al. Quantification of extracellular volume fraction by cardiac computed tomography for noninvasive assessment of myocardial fibrosis in hemodialysis patients. Sci Rep. 2020;10:15367. 32958834 10.1038/s41598-020-72417-5 PMC7506012

[40] Hoffmann U, et al. Noninvasive assessment of plaque morphology and composition in culprit and stable lesions in acute coronary syndrome and stable lesions in stable angina by multidetector computed tomography. J Am Coll Cardiol. 2006;47:1655–62. 16631006 10.1016/j.jacc.2006.01.041

[41] Andreini D, et al. Coronary In-Stent Restenosis: Assessment with CT Coronary Angiography. Radiology. 2012;265:410–7. 22966068 10.1148/radiol.12112363

[42] Lubbers M, et al. Calcium imaging and selective computed tomography angiography in comparison to functional testing for suspected coronary artery disease: the multicentre, randomized CRESCENT trial. Eur Heart J. 2016;37:1232–43. 26746631 10.1093/eurheartj/ehv700

[43] Gassert FG, et al. Calcium scoring using virtual non-contrast images from a dual-layer spectral detector CT: comparison to true non-contrast data and evaluation of proportionality factor in a large patient collective. Eur Radiol. 2021;31:6193–9. 33474570 10.1007/s00330-020-07677-w PMC8270810

[44] Otton JM, et al. A method for coronary artery calcium scoring using contrast-enhanced computed tomography. J Cardiovasc Comput Tomogr. 2012;6:37–44. 22210533 10.1016/j.jcct.2011.11.004

[45] Tonino PAL, et al. Fractional Flow Reserve versus Angiography for Guiding Percutaneous Coronary Intervention. N Engl J Med. 2009;360:213–24. 19144937 10.1056/NEJMoa0807611

[46] Davies JE, et al. Use of the Instantaneous Wave-free Ratio or Fractional Flow Reserve in PCI. N Engl J Med. 2017;376:1824–34. 28317458 10.1056/NEJMoa1700445

[47] Knuuti J, et al. 2019 ESC Guidelines for the diagnosis and management of chronic coronary syndromes: The Task Force for the diagnosis and management of chronic coronary syndromes of the European Society of Cardiology (ESC). Eur Heart J. 2020;41:407–77. 31504439 10.1093/eurheartj/ehz425

[48] Hecht HS, Narula J, Fearon WF. Fractional Flow Reserve and Coronary Computed Tomographic Angiography. Circ Res. 2016;119:300–16. 27390333 10.1161/CIRCRESAHA.116.307914

[49] Agasthi P, et al. Comparison of Computed Tomography derived Fractional Flow Reserve to invasive Fractional Flow Reserve in Diagnosis of Functional Coronary Stenosis: A Meta-Analysis. Sci Rep. 2018;8:11535. 30069020 10.1038/s41598-018-29910-9 PMC6070545

[50] Rajiah P, Cummings KW, Williamson E, Young PM. CT Fractional Flow Reserve: A Practical Guide to Application, Interpretation, and Problem Solving. Radiographics. 2022;42:340–58. 35119968 10.1148/rg.210097

[51] Kurata A, et al. Myocardial Perfusion Imaging Using Adenosine Triphosphate Stress Multi-Slice Spiral Computed Tomography Alternative to Stress Myocardial Perfusion Scintigraphy: Alternative to Stress Myocardial Perfusion Scintigraphy. Circ J. 2005;69:550–7. 15849441 10.1253/circj.69.550

[52] George RT, et al. Multidetector computed tomography myocardial perfusion imaging during adenosine stress. J Am Coll Cardiol. 2006;48:153–60. 16814661 10.1016/j.jacc.2006.04.014

[53] Otton J, et al. A direct comparison of the sensitivity of CT and MR cardiac perfusion using a myocardial perfusion phantom. J Cardiovasc Comput Tomogr. 2013;7:117–24. 23622506 10.1016/j.jcct.2013.01.016 PMC3994525

[54] Ko BS, et al. Computed tomography stress myocardial perfusion imaging in patients considered for revascularization: a comparison with fractional flow reserve. Eur Heart J. 2012;33:67–77. 21810860 10.1093/eurheartj/ehr268

[55] Bettencourt N, et al. Direct Comparison of Cardiac Magnetic Resonance and Multidetector Computed Tomography Stress-Rest Perfusion Imaging for Detection of Coronary Artery Disease. J Am Coll Cardiol. 2013;61:1099–107. 23375929 10.1016/j.jacc.2012.12.020

[56] Bettencourt N, Ferreira ND, Leite D, Carvalho M, Ferreira WDS, Schuster A, et al. CAD detection in patients with intermediate-high pre-test probability: low-dose CT delayed enhancement detects ischemic myocardial scar with moderate accuracy but does not improve performance of a stress-rest CT perfusion protocol. JACC Cardiovasc Imaging. 2013;6:1062–71. 10.1016/j.jcmg.2013.04.013 24011773

[57] Juergens KU, et al. Multi-Detector Row CT of Left Ventricular Function with Dedicated Analysis Software versus MR Imaging: Initial Experience. Radiology. 2004;230:403–10. 14668428 10.1148/radiol.2302030042

[58] Sugeng L, et al. Quantitative assessment of left ventricular size and function: side-by-side comparison of real-time three-dimensional echocardiography and computed tomography with magnetic resonance reference. Circulation. 2006;114:654–61. 16894035 10.1161/CIRCULATIONAHA.106.626143

[59] Asferg C, Usinger L, Kristensen TS, Abdulla J. Accuracy of multi-slice computed tomography for measurement of left ventricular ejection fraction compared with cardiac magnetic resonance imaging and two-dimensional transthoracic echocardiography: A systematic review and meta-analysis. Eur J Radiol. 2012;81:e757–62. 22381439 10.1016/j.ejrad.2012.02.002

[60] Guo Y, et al. Accuracy and reproducibility of assessing right ventricular function with 64-section multi-detector row CT: Comparison with magnetic resonance imaging. Int J Cardiol. 2010;139:254–62. 19028401 10.1016/j.ijcard.2008.10.031

[61] McVeigh ER, et al. Regional myocardial strain measurements from 4DCT in patients with normal LV function. J Cardiovasc Comput Tomogr. 2018;12:372–8. 29784623 10.1016/j.jcct.2018.05.002 PMC7458583

[62] Wang R, et al. Quantitative analysis of three-dimensional left ventricular global strain using coronary computed tomography angiography in patients with heart failure: Comparison with 3T cardiac MR. Eur J Radiol. 2021;135:109485. 33401113 10.1016/j.ejrad.2020.109485

[63] Mahnken AH, Bruners P, Bornikoel CM, Krämer N, Guenther RW. Assessment of Myocardial Edema by Computed Tomography in Myocardial Infarction. JACC Cardiovasc Imaging. 2009;2:1167–74. 19833305 10.1016/j.jcmg.2009.05.014

[64] van Assen M, et al. Feasibility of extracellular volume quantification using dual-energy CT. J Cardiovasc Comput Tomogr. 2019;13:81–4. 30377090 10.1016/j.jcct.2018.10.011

[65] Nacif MS, et al. Interstitial myocardial fibrosis assessed as extracellular volume fraction with low-radiation-dose cardiac CT. Radiology. 2012;264:876–83. 22771879 10.1148/radiol.12112458 PMC3426854

[66] Prevrhal S, Forsythe CH, Harnish RJ, Saeed M, Yeh BM. CT angiographic measurement of vascular blood flow velocity by using projection data. Radiology. 2011;261:923–9. 21969665 10.1148/radiol.11110617

[67] Franke B et al. (2021) Computed Tomography-Based Assessment of Transvalvular Pressure Gradient in Aortic Stenosis. Front. Cardiovasc. Med. 8:706628. doi: 10.3389/fcvm.2021.706628 10.3389/fcvm.2021.706628 PMC8457381 34568450

[68] Mittal R, et al. Computational modeling of cardiac hemodynamics: Current status and future outlook. J Comput Phys. 2016;305:1065–82.

[69] Lantz J, Henriksson L, Persson A, Karlsson M, Ebbers T. Patient-Specific Simulation of Cardiac Blood Flow From High-Resolution Computed Tomography. J Biomech Eng. 2016;138(12):121004. 10.1115/1.4034652 27618494

[70] Bergström G, et al. Prevalence of Subclinical Coronary Artery Atherosclerosis in the General Population. Circulation. 2021;144:916–29. 34543072 10.1161/CIRCULATIONAHA.121.055340 PMC8448414

[71] Carr JJ, et al. Association of Coronary Artery Calcium in Adults Aged 32 to 46 Years With Incident Coronary Heart Disease and Death. JAMA Cardiol. 2017;2:391–9. 28196265 10.1001/jamacardio.2016.5493 PMC5397328

[72] Arnett DK, et al. 2019 ACC/AHA Guideline on the Primary Prevention of Cardiovascular Disease: A Report of the American College of Cardiology/American Heart Association Task Force on Clinical Practice Guidelines. Circulation. 2019;140:e596–646. 30879355 10.1161/CIR.0000000000000678 PMC7734661

[73] Chang H-J, et al. Coronary Atherosclerotic Precursors of Acute Coronary Syndromes. J Am Coll Cardiol. 2018;71:2511–22. 29852975 10.1016/j.jacc.2018.02.079 PMC6020028

[74] Lin FY, et al. Mortality Risk in Symptomatic Patients With Nonobstructive Coronary Artery Disease: A Prospective 2-Center Study of 2,583 Patients Undergoing 64-Detector Row Coronary Computed Tomographic Angiography. J Am Coll Cardiol. 2011;58:510–9. 21777749 10.1016/j.jacc.2010.11.078

[75] Detrano R, et al. Coronary Calcium as a Predictor of Coronary Events in Four Racial or Ethnic Groups. N Engl J Med. 2008;358:1336–45. 18367736 10.1056/NEJMoa072100

[76] Budoff MJ, et al. Coronary Calcium Predicts Events Better With Absolute Calcium Scores Than Age-Sex-Race/Ethnicity Percentiles: MESA (Multi-Ethnic Study of Atherosclerosis). J Am Coll Cardiol. 2009;53:345–52. 19161884 10.1016/j.jacc.2008.07.072 PMC2652569

[77] Mortensen MB, et al. Impact of Plaque Burden Versus Stenosis on Ischemic Events in Patients With Coronary Atherosclerosis. J Am Coll Cardiol. 2020;76:2803–13. 33303068 10.1016/j.jacc.2020.10.021

[78] van Rosendael AR, et al. Association of Statin Treatment With Progression of Coronary Atherosclerotic Plaque Composition. JAMA Cardiol. 2021;6:1257–66. 34406326 10.1001/jamacardio.2021.3055 PMC8374741

[79] Mitchell JD, et al. Impact of Statins on Cardiovascular Outcomes Following Coronary Artery Calcium Scoring. J Am Coll Cardiol. 2018;72:3233–42. 30409567 10.1016/j.jacc.2018.09.051 PMC6309473

[80] Greenland P, Blaha MJ, Budoff MJ, Erbel R, Watson KE. Coronary Calcium Score and Cardiovascular Risk. J Am Coll Cardiol. 2018;72:434–47. 30025580 10.1016/j.jacc.2018.05.027 PMC6056023

[81] De Bosscher R, et al. Lifelong endurance exercise and its relation with coronary atherosclerosis. Eur Heart J. 2023;44:2388–99. 36881712 10.1093/eurheartj/ehad152 PMC10327878

[82] Recent-onset chest pain of suspected cardiac origin: assessment and diagnosis. London: National Institute for Health and Care Excellence (NICE); 2016 Nov 30. (NICE Clinical Guidelines, No. 95.) Available from: https://www.ncbi.nlm.nih.gov/books/NBK553650/32065740

[83] Fihn SD, et al. 2012 ACCF/AHA/ACP/AATS/PCNA/SCAI/STS guideline for the diagnosis and management of patients with stable ischemic heart disease: executive summary: a report of the American College of Cardiology Foundation/American Heart Association task force on practice guidelines, and the American College of Physicians, American Association for Thoracic Surgery, Preventive Cardiovascular Nurses Association, Society for Cardiovascular Angiography and Interventions, and Society of Thoracic Surgeons. Circulation. 2012;126:3097–137. 23166210 10.1161/CIR.0b013e3182776f83

[84] Collet J-P, et al. 2020 ESC Guidelines for the management of acute coronary syndromes in patients presenting without persistent ST-segment elevation: The Task Force for the management of acute coronary syndromes in patients presenting without persistent ST-segment elevation of the European Society of Cardiology (ESC). Eur Heart J. 2021;42:1289–367. 32860058 10.1093/eurheartj/ehaa575

[85] Knuuti J, et al. The performance of non-invasive tests to rule-in and rule-out significant coronary artery stenosis in patients with stable angina: a meta-analysis focused on post-test disease probability. Eur Heart J. 2018;39:3322–30. 29850808 10.1093/eurheartj/ehy267

[86] Budoff MJ, et al. Prognostic Value of Coronary Artery Calcium in the PROMISE Study (Prospective Multicenter Imaging Study for Evaluation of Chest Pain). Circulation. 2017;136:1993–2005. 28847895 10.1161/CIRCULATIONAHA.117.030578 PMC5698136

[87] SCOT-HEART Investigators, et al. Coronary CT Angiography and 5-Year Risk of Myocardial Infarction. N Engl J Med. 2018;379:924–33. 30145934 10.1056/NEJMoa1805971

[88] Villines TC, et al. Prevalence and severity of coronary artery disease and adverse events among symptomatic patients with coronary artery calcification scores of zero undergoing coronary computed tomography angiography: results from the CONFIRM (Coronary CT Angiography Evaluation for Clinical Outcomes: An International Multicenter) registry. J Am Coll Cardiol. 2011;58:2533–40. 22079127 10.1016/j.jacc.2011.10.851

[89] Maurovich-Horvat, P.; Bosserdt, M.; Kofoed, K.F.; Rieckmann, N.; Benedek, T.; Donnelly, P.; Rodriguez-Palomares, J.; Erglis, A.; Štěchovský, C.; Šakalyte, G. CT or invasive coronary angiography in stable chest pain. N. Engl. J. Med. 2022, 386, 1591–1602 10.1056/NEJMoa2200963 35240010

[90] Klüner LV, Oikonomou EK, Antoniades C. Assessing Cardiovascular Risk by Using the Fat Attenuation Index in Coronary CT Angiography. Radiol Cardiothorac Imaging. 2021;3:e200563. 33778665 10.1148/ryct.2021200563 PMC7977699

[91] Agus AM, et al. The cost-effectiveness of cardiac computed tomography for patients with stable chest pain. Heart Br Card Soc. 2016;102:356–62. 10.1136/heartjnl-2015-308247 26769552

[92] Halpern EJ, Savage MP, Fischman DL, Levin DC. Cost-effectiveness of coronary CT angiography in evaluation of patients without symptoms who have positive stress test results. AJR Am J Roentgenol. 2010;194:1257–62. 20410412 10.2214/AJR.09.3209

[93] Cury RC, et al. CAD-RADS^TM^ 2.0 - 2022 Coronary Artery Disease-Reporting and Data System: An Expert Consensus Document of the Society of Cardiovascular Computed Tomography (SCCT), the American College of Cardiology (ACC), the American College of Radiology (ACR), and the North America Society of Cardiovascular Imaging (NASCI). J Cardiovasc Comput Tomogr. 2022;16:536–57. 35864070 10.1016/j.jcct.2022.07.002

[94] Goldstein JA, et al. The CT-STAT (Coronary Computed Tomographic Angiography for Systematic Triage of Acute Chest Pain Patients to Treatment) trial. J Am Coll Cardiol. 2011;58:1414–22. 21939822 10.1016/j.jacc.2011.03.068

[95] Litt HI, et al. CT Angiography for Safe Discharge of Patients with Possible Acute Coronary Syndromes. N Engl J Med. 2012;366:1393–403. 22449295 10.1056/NEJMoa1201163

[96] Hoffmann U, et al. Coronary CT Angiography versus Standard Evaluation in Acute Chest Pain. N Engl J Med. 2012;367:299–308. 22830462 10.1056/NEJMoa1201161 PMC3662217

[97] Nørgaard BL, et al. Coronary CT Angiography-derived Fractional Flow Reserve Testing in Patients with Stable Coronary Artery Disease: Recommendations on Interpretation and Reporting. Radiol Cardiothorac Imaging. 2019;1:e190050. 33778528 10.1148/ryct.2019190050 PMC7977999

[98] Leipsic J, et al. SCCT guidelines for the interpretation and reporting of coronary CT angiography: a report of the Society of Cardiovascular Computed Tomography Guidelines Committee. J Cardiovasc Comput Tomogr. 2014;8:342–58. 25301040 10.1016/j.jcct.2014.07.003

[99] Min JK, et al. Diagnostic accuracy of fractional flow reserve from anatomic CT angiography. JAMA. 2012;308:1237–45. 22922562 10.1001/2012.jama.11274 PMC4281479

[100] Wang Z-Q, et al. Diagnostic accuracy of a deep learning approach to calculate FFR from coronary CT angiography. J Geriatr Cardiol JGC. 2019;16:42–8. 30800150 10.11909/j.issn.1671-5411.2019.01.010 PMC6379239

[101] Gonzalez JA, et al. Meta-Analysis of Diagnostic Performance of Coronary Computed Tomography Angiography, Computed Tomography Perfusion, and Computed Tomography-Fractional Flow Reserve in Functional Myocardial Ischemia Assessment Versus Invasive Fractional Flow Reserve. Am J Cardiol. 2015;116:1469–78. 26347004 10.1016/j.amjcard.2015.07.078 PMC4851492

[102] Curzen N, et al. Fractional flow reserve derived from computed tomography coronary angiography in the assessment and management of stable chest pain: the FORECAST randomized trial. Eur Heart J. 2021;42:3844–52. 34269376 10.1093/eurheartj/ehab444 PMC8648068

[103] Maffei E, et al. Left and right ventricle assessment with Cardiac CT: validation study vs. Cardiac MR Eur Radiol. 2012;22:1041–9. 22270140 10.1007/s00330-011-2345-6 PMC3321142

[104] Rahaghi FN, et al. Ventricular geometry from non-contrast non-ECG gated CT scans: an imaging marker of cardiopulmonary disease in smokers. Acad Radiol. 2017;24:594–602. 28215632 10.1016/j.acra.2016.12.007 PMC5653289

[105] Shahzad R, et al. Automatic segmentation and quantification of the cardiac structures from non-contrast-enhanced cardiac CT scans. Phys Med Biol. 2017;62:3798–813. 28248196 10.1088/1361-6560/aa63cb

[106] Fredgart MH, et al. Measurement of left atrial volume by 2D and 3D non-contrast computed tomography compared with cardiac magnetic resonance imaging. J Cardiovasc Comput Tomogr. 2018;12:316–9. 29666031 10.1016/j.jcct.2018.04.001

[107] Liu S, et al. Diffuse myocardial fibrosis evaluation using cardiac magnetic resonance T1 mapping: sample size considerations for clinical trials. J Cardiovasc Magn Reson. 2012;14:90. 23272704 10.1186/1532-429X-14-90 PMC3552738

[108] Ternacle J, et al. Aortic Stenosis and Cardiac Amyloidosis: JACC Review Topic of the Week. J Am Coll Cardiol. 2019;74:2638–51. 31753206 10.1016/j.jacc.2019.09.056

[109] Scully PR, et al. Identifying Cardiac Amyloid in Aortic Stenosis: ECV Quantification by CT in TAVR Patients. JACC Cardiovasc Imaging. 2020;13:2177–89. 32771574 10.1016/j.jcmg.2020.05.029 PMC7536272

[110] Ommen SR, et al. 2020 AHA/ACC Guideline for the Diagnosis and Treatment of Patients With Hypertrophic Cardiomyopathy: A Report of the American College of Cardiology/American Heart Association Joint Committee on Clinical Practice Guidelines. J Am Coll Cardiol. 2020;76:e159–240. 33229116 10.1016/j.jacc.2020.08.045

[111] Te Riele ASJM, Tandri H, Sanborn DM, Bluemke DA. Noninvasive Multimodality Imaging in ARVD/C. JACC Cardiovasc Imaging. 2015;8:597–611. 25937197 10.1016/j.jcmg.2015.02.007 PMC4755585

[112] Vahanian A, et al. 2021 ESC/EACTS Guidelines for the management of valvular heart disease: Developed by the Task Force for the management of valvular heart disease of the European Society of Cardiology (ESC) and the European Association for Cardio-Thoracic Surgery (EACTS). Eur Heart J. 2022;43:561–632. 34453165 10.1093/eurheartj/ehab395

[113] Evertz R, Hub S, Kowallick JT, Seidler T, Danner BC, Hasenfuß G, et al. Impact of observer experience on multidetector computed tomography aortic valve morphology assessment and valve size selection for transcatheter aortic valve replacement. Sci Rep. 2022;12:21430. 10.1038/s41598-022-23936-w PMC9744877 36509862

[114] Evertz R, Hub S, Backhaus SJ, Lange T, Toischer K, Kowallick JT, et al. Head-to-Head Comparison of Different Software Solutions for AVC Quantification Using Contrast-Enhanced MDCT. J Clin Med. 2021;10:3970. 10.3390/jcm10173970 PMC8432112 34501418

[115] Evertz R, Hub S, Beuthner BE, Backhaus SJ, Lange T, Schulz A, et al. Aortic valve calcification and myocardial fibrosis determine outcome following transcatheter aortic valve replacement. ESC Heart Fail. 2023;10:2307–18. 10.1002/ehf2.14307 PMC10375183 37060191

[116] Hansson NC, et al. Aortic valve and left ventricular outflow tract calcium volume and distribution in transcatheter aortic valve replacement: Influence on the risk of significant paravalvular regurgitation. J Cardiovasc Comput Tomogr. 2018;12:290–7. 29519754 10.1016/j.jcct.2018.02.002

[117] Barbanti M, et al. Anatomical and procedural features associated with aortic root rupture during balloon-expandable transcatheter aortic valve replacement. Circulation. 2013;128:244–53. 23748467 10.1161/CIRCULATIONAHA.113.002947

[118] Yoon S-H, et al. Predictors of Left Ventricular Outflow Tract Obstruction After Transcatheter Mitral Valve Replacement. JACC Cardiovasc Interv. 2019;12:182–93. 30678797 10.1016/j.jcin.2018.12.001

[119] Pontone G, et al. Clinical applications of cardiac computed tomography: a consensus paper of the European Association of Cardiovascular Imaging-part II. Eur Heart J Cardiovasc Imaging. 2022;23:e136–61. 35175348 10.1093/ehjci/jeab292 PMC8944330

[120] Schulz A, Beuthner BE, Böttiger ZM, Gersch SS, Lange T, Gronwald J, et al. Epicardial adipose tissue as an independent predictor of long-term outcome in patients with severe aortic stenosis undergoing transcatheter aortic valve replacement. Clin Res Cardiol. 2024. 10.1007/s00392-024-02387-5. 10.1007/s00392-024-02387-5 PMC12540550 38324040

[121] Delgado V, et al. 2023 ESC Guidelines for the management of endocarditis: Developed by the task force on the management of endocarditis of the European Society of Cardiology (ESC) Endorsed by the European Association for Cardio-Thoracic Surgery (EACTS) and the European Association of Nuclear Medicine (EANM). Eur Heart J. 2023;44:3948–4042. 37738322 10.1093/eurheartj/ehad625

[122] Nagiub M, et al. Value of Time-Resolved Cardiac CT in Children and Young Adults with Congenital Heart Disease and Infective Endocarditis. Pediatr Cardiol. 2022. 10.1007/s00246-022-03069-7. 36534136 10.1007/s00246-022-03069-7

[123] Hendriks BMF, Martens B, Mihl C. Pre-procedural computed tomography in transcatheter pulmonary valve replacement: The first steps towards standardization of image quality. Int J Cardiol Heart Vasc. 2020;29:100542. 32885028 10.1016/j.ijcha.2020.100542 PMC7452673

[124] Ferraro AM, et al. Computed tomography angiography (CTA) of anomalous aortic origin of a coronary artery (AAOCA): Which measurements are accurate and reliable? J Cardiovasc Comput Tomogr. 2023;17:130–7. 36804387 10.1016/j.jcct.2023.02.003

[125] Otton JM, et al. 3D printing from cardiovascular CT: a practical guide and review. Cardiovasc Diagn Ther. 2017;7:507–26. 29255693 10.21037/cdt.2017.01.12 PMC5716949

